# Supplementary material for: Human Vitamin K Epoxide Reductase as a Target of Its Redox Protein
Source: Int J Mol Sci. 2022 Mar 31;23(7):3899. doi: 10.3390/ijms23073899 (PMC8998853; doi:10.3390/ijms23073899)
Supplement: Supplementary file 1 [file ijms-23-03899-s001.zip › ijms-1658853-supplementary.pdf]

# Supplementary material

## Human Vitamin K Epoxide Reductase as a Target of its Redox Protein

Julie Ledoux[1#], Maxim Stolyarchuk[1#], Enki Bachelier[1],

Alain Trouvé and Luba Tchertanov[1*]

[1] Centre Borelli, ENS Paris-Saclay, CNRS, Université Paris-Saclay, 4 avenue des Sciences, F-91190 Gif-sur-Yvette, France

**Table S1. Hydrogen bonds involving L-loop residues in crystallographic structures 6wvi (apo-c) and 6wv3 (holo-c) of VKOR in the oxidized state.** Distances (D···A and H···A, Å) between donor (D) and acceptor (A) atoms, and hydrogen (H) and acceptor (A) atoms, and the pseudo-valent angle at H (°). Amino acids forming H-bond by backbone and side chains are denoted in black and blue respectively. H-bonds observed in both forms are highlighted in blue.

| H-bond | Distance D...A/H...A/ ∠A–H–D | H-bond | Distance D...A/H...A/ ∠A–H–D |
|---|---|---|---|
| apo-c | | holo-c | |
| D36(N)...H33(O) | 3.09/2.16/157.8 | D36(N)...A32(O) | 2.83/1.87/157.6 |
| Y39(O)...S50(O) | 2.47 /1.54/167.4 | R33(N)...R37(O) | 2.84/1.88/158.8 |
| S50(N)...S48(O) | 3.12/2.24/163.3 | R40(N)...G46(O) | 2.93/1.97/156.3 |
| S52(N)...Y39(O) | 3.12/2.23/149.9 | L42(N)...Y25(O;TM1) | 2.90/1.96/153.7 |
| R61(N)...N77(O) | 3.06/2.14/154.4 | D44(N)...A41(O) | 3.01/2.08/153.2 |
| | | I49 (O2)... V45(N) | 3.05/2.20/140.5 |
| G62(N)...Q78(O) | 2.65/1.72/156.0 | G62(N)...Q78(O2) | 2.72/1.84/143.4 |
| F63(N)...N80(O;TM2) | 2.85/2.02/139.8 | F63(N)...N80(O;TM2) | 2.87/1.91/156.7 |
| S68(N)...F70(O) | 2.77/2.07/126.4 | L76(N)...S74(O) | 2.97/2.06/148.3 |
| D73(N)...G71(O) | 3.00/2.25/131.5 | | |
| Q78(N)...A75(O) | 2.57/1.62/158.4 | Q78(N)...I75(O) | 3.05/2.13/150.5 |
| N80(N;TM2)...W59(O) | 2.58/1.69/148.0 | N80(N;TM2)...W59(O) | 2.79/1.81/164.4 |
| N80(N;TM2)...G60(O) | 2.96/2.06/150.0 | N80(N;TM2)...G60(O) | 3.18/2.19/166.8 |

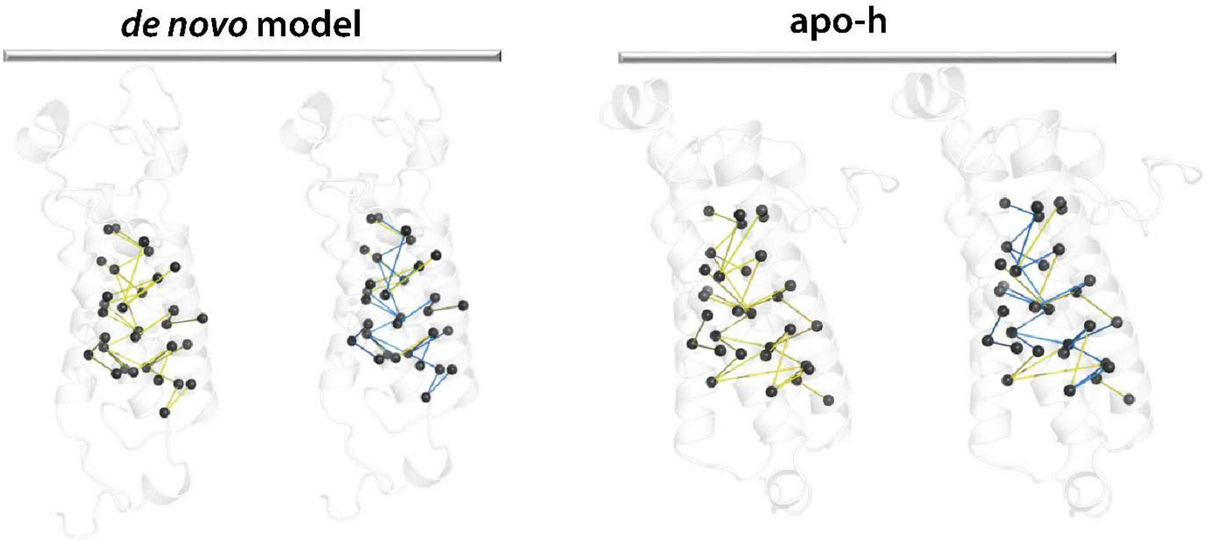


**Figure S1**. Non-covalent interactions stabilising hVKORC1 TMD helices in *de novo* model embedded in phospholipid bilayers mimicking the ER membrane in aqueous solution, and apo-h form simulated in aqueous solution. Non-covalent interactions, denoted as an undirected graph whose vertices represent a residue and a link between two residues reflects the presence of at least one type of non-covalent interaction. Van-der-Waals contacts (≤4.0 Å, events with frequency ≥ 0.8) were calculated for all heavy atoms (O, N, C and S). All observed contacts in each model (left) and blue links discriminated contacts common between two models (right).

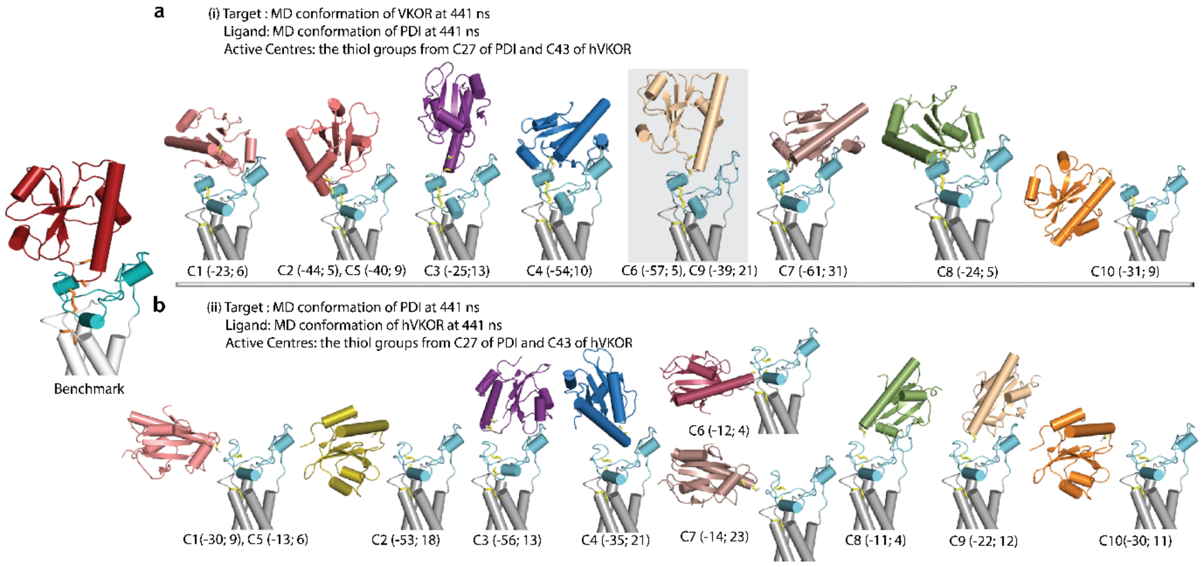


**Figure S2**. Protein-protein computational docking using an information–driven method on benchmark complex PDI-hVKORC1. Top 10 models produced by HADDOCK for the reference structure (benchmark) using two scenarios for a ligand-target pair, (i) (**a**) and (ii) (**b**). For each cluster, the representative conformations with the HADDOCK score (a.u.) and a cluster population (number of observation) are shown. Protein is shown as a cartoon with helices as cylinders and disulfide bridges in yellow sticks.
